# miR-450b-3p inhibited the proliferation of gastric cancer via regulating KLF7

**DOI:** 10.1186/s12935-020-1133-2

**Published:** 2020-02-10

**Authors:** Juan Yao, Hao Zhang, Cheng Liu, Shuangshuang Chen, Rongyu Qian, Kun Zhao

**Affiliations:** 1 Department of Oncology, Taizhou People’s Hospital Affiliated to Nantong University, Taizhou, Jiangsu China; 2 Department of Interventional Radiology, Huaian Hospital of Huaian City, No. 161 Zhenhuailou East Road, Huai’an, 223200 Jiangsu People’s Republic of China; 3 Department of Oncology, Huaian Hospital of Huaian City, No. 161 Zhenhuailou East Road, Huai’an, 223200 Jiangsu People’s Republic of China

**Keywords:** miR-450b-3p, KLF7, Gastric cancer, Proliferation

## Abstract

**Background:**

This study aimed to investigate the clinical characteristics of miR-450b-3p in the patients of gastric cancer (GC), and further explore whether miR-450b-3p could inhibit the proliferation of GC cells via regulating KLF7.

**Methods:**

Real-time quantitative PCR (qRT-PCR) was performed to detect the expression level of miR-450b-3p in 48 GC patients of tumor tissue and paracancerous tissue specimens collected, and the associations between miR-450b-3p and the clinical characteristics of GC patients were analyzed. Meanwhile, the expression of miR-450b-3p in GC cell lines was verified using qRT-PCR. miR-450b-3p overexpression vectors was constructed in GC cell lines including AGS and BGC-823, and then CCK-8 cell proliferation assay, Plate colony formation assay and EdU assay were applied to analyze the biological function of miR-450b-3p in GC cell lines.

**Results:**

The results of qRT-PCR showed that the expression level of miR-450b-3p in GC tissues was lower than that in paracancerous tissues, and the difference was statistically significant. Compared with GC patients with high-miR-450b-3p expression, these GC patients with low-miR-450b-3p expression had a higher pathological stage and tumor size. Subsequently, the proliferation ability of GC cells in miR-450b-3p mimic was significantly decreased when comparing with the NC mimic. In addition, qRT-PCR indicated that the expression level of KLF7 significantly decreased after miR-450b-3p mimic. Therefore, it was demonstrated that miR-450b-3p might inhibit the malignant progression of GC via modulating KLF7. Bioinformatics analysis and dual luciferase reporter suggested miR-450b-3p was bound to KLF7. Finally, the results of the reverse experiment confirmed that overexpression of KLF7 could reverse miR-450b-3p mimic induced-inhibition of GC malignant progression.

**Conclusions:**

Generally, miR-450b-3p significantly down-regulated in GC tissues and cell lines, and was associated with the pathological stage and tumor size of GC patients. Meanwhile, miR-450b-3p inhibited cell proliferation in GC via modulating KLF7.

## Background

Gastric cancer (GC) is the most common malignant tumor of the digestive system, with its mortality the second among all cancer in the world [1, 2]. Due to the lack of the early screening of GC, the diagnosis of GC is mostly confirmed in an advanced stage, and the 5-year survival rate after radical gastrectomy is only about 40% in China [3, 4]. The pathogenesis of GC is a complex multi-step and multi-stage process, involving multiple genes and environment factors [5]. Thus, the molecular targeted therapy of GC is gradually applied in clinical practice [6, 7]. However, how to further improve the therapeutic effect of molecular targeted drugs in advanced GC is the current problems [7]. Therefore, it is necessary to find valuable diagnostic and prognostic biomarkers of GC, thus improving the premise of molecular targeted therapy as well as understanding the potential regulation mechanism [8, 9].

A large number of studies have shown that the occurrence of GC was related to genetic or epigenetic changes [10, 11]. Recently, non-coding RNA (ncRNA), including microRNA (miRNA) in the progression of GC has attracted the attention of the researchers [11, 12]. MiRNAs are a class of endogenous non-coding RNA (about 19–24 nucleotides in length) that regulate gene expression through complete or incomplete pairing with complementary nucleotide sequences of the target mRNA 3′-terminal non-translation region (3′-UTR) after transcription [13, 14]. These miRNA networks regulate the expression of more than 30% of cellular proteins [15]. Previous studies have shown that miRNAs could be involved in a wide range of cell biological processes, including cell proliferation, differentiation, apoptosis, and metastasis and so on [14, 16]. It has been reported that miR-450b-3p was down-regulated in several tumors, serving as a tumor suppressor in the malignant progression [17, 18].

Bioinformatics analysis speculated that miR-450b-3p might target Krüppel-like factor 7 (KLF7). KLF7, also referred to as ubiquitous Krüppel-like factor, is expressed at high levels in numerous human tissues [19, 20]. In addition, KLF7 could effectively destroy the histological barrier that blocks tumor, and eventually cause tumor cells to detach from the primary site with complete structure to damage surrounding tissues [20]. Based on above, this study intended to investigate the biological function of miR-450b-3p in the malignant progression of GC, as well as analyze the correlations between the expression of miR-450b-3p and the clinical characteristics of GC patients. Besides, we also need to further explore whether miR-450b-3p inhibited the proliferation of GC cells through the regulation of KLF7, so as to provide a new target for the diagnosis and treatment of GC.

## Materials and methods

### Patients and GC samples

In this study, 48 pairs of GC tumor tissue samples and corresponding paracancerous tissue ones were selected from surgically treated GC patients and then stored at − 80 °C. The study was approved by the Ethics Committee, and all participating patients and healthy volunteers had fully signed the informed consent, to agree that these specimens would be used for scientific researches.

### Cell lines and reagents

Human GC cell lines (AGS, BGC-823, SGC-7901, MKN28 and MKN45) and human normal gastric mucosal epithelial cell line (GES-1) were purchased from American ATCC (American rype Culture Collection). All these cell lines were cultured with DMEM (Dulbecco’s Modified MEM medium) high glucose medium containing 10% fetal calf serum, penicillin (100 U/mL) and streptomycin (100 ug/mL) in a 37 °C, 5% CO_2_ incubator. Cells were passaged with 1% trypsin + EDTA for digestion when grown to 80–90% confluence.

### Transfection

The negative control (NC mimic) and the miR-450b-3p overexpression vectors (miR-450b-3p mimic) were purchased from GenePharma (GenePharma, Shanghai, China). Cells were plated in 6-well plates and grown to a cell density of 40%, and then transfection was performed according to the manufacturer’s instructions. After 48 h, cells were collected for qRT-PCR analysis and function experiments.

### CCK8 assay

The cells after 48 h of transfection were harvested and were plated into 96-well plates at 2000 cells per well. After cultured for 24 h, 48 h, 72 h and 96 h, these cells were added with CCK-8 Kit (Dojindo Laboratories, Japan). After incubation for 2 h, the fluorescent absorbance was measured at the optical density (OD) value of 570 nm each well in the microplate reader.

### Colony formation assay

Cells were seeded in the 6-well plate with 2.5 × 10^3^ cells per well and cultured for 2 weeks. Subsequently, cells were subjected to 15-min fixation in 4% paraformaldehyde and 10-min staining in Giemsa solution. After the methanol was aspirated, the cells were stained with 0.1% crystal violet staining solution for 20 min, washed 3 times with PBS, photographed and counted under a light-selective environment.

### 5-ethynyl-29-deoxyuridine (EdU) assay

EdU proliferation assay (RiboBio, Nanjing, China) was performed according to the manufacturer’s requirements. After transfection for 24 h, the cells were incubated with 50 μM EdU for 2 h, then stained with AdoLo and DAPI, and the number of EdU-positive cells was detected by fluorescence microscopy. The display rate of EdU positive is shown as the ratio of the number of EdU positive cells to the total DAPI chromogenic cells (blue cells).

### Quantitative real-time PCR (qRT-PCR)

After the cells were treated accordingly, 1 ml of Trizol was used to lyse the cells, and total RNA was extracted. The initially extracted RNA was treated with DNase I to remove genomic DNA and repurify the RNA. RNA reverse transcription was performed according to the Prime Scirpt Reverse Transcription Kit (Takara) instructions, real-time PCR was performed according to the SYBR^®^ Premix Ex TaqTM (Takara) kit instructions, and the PCR reaction was performed using the StepOne Plus Real-time PCR System (Applied Biosystems, Foster City, CA, USA). Three replicate wells were repeated for each sample and the assay was repeated twice. The Bio-Rad PCR instrument was used to analyze and process the data with the software iQ5 2.0. The GAPDH and U6 genes were used as internal parameters, and the gene expression was calculated by the 2^−ΔΔCt^ method. The following primers were used for qRT-PCR reactions:

miR-450b-3p:

forward, 5′-GATCCCCGGAUGCAAAAUGAUCCCAATTCA-3′,

reverse, 5′-AGCTTAAAAAGGAUGCAAAAUGAUCCCAAT-3′;

U6:

forward, 5′-CTCGCTTCGGCAGCACA-3′,

reverse, 5′-AACGCTTCACGAATTTGCGT-3′;

KLF7:

forward, 5′-ACTGCTTGCTGACAATCTCG-3′,

reverse, 5′-GGTCCCTCACACATCCTTCA-3′;

GAPDH:

forward, 5′-ACAACTTTGGTATCGTGGAAGG-3′,

reverse, 5′-GCCATCACGCCACAGTTTC-3′.

### Western blotting

The transfected cells were lysed using PRO-PREPTM lysis buffer, shaken on ice for 30 min, and centrifuged at 14,000×*g* for 15 min at 4 °C. Total protein concentration was calculated by the GCA Protein Assay Kit (Pierce, Rockford, Il, USA). Rabbit anti-human monoclonal antibodies against KLF7 were purchased from Santa Cruz, USA; horseradish peroxidase-labeled goat anti-rabbit secondary antibody was purchased from Genscript. GAPDH was used as the internal reference control. Protein samples were separated by SDS-PAGE, transferred to PVDF membrane, and blocked with 5% skim milk powder for 1 h at room temperature. Primary antibodies were added for incubation overnight at 4 °C shaker. In the next day, the membrane was rinsed 3 times with TBST and incubated with second antibody for 1 h at room temperature. After that, the protein samples on the membrane were finally semi-quantitatively analyzed by alpha SP image analysis software.

### Dual-luciferase reporter assay

3′-UTR of wild-type (WT) human KLF7 gene, which contains a putative miR-450b-3p binding DNA sequence, was amplified by PCR and inserted into a p-miR-reporter (Ambion, USA) to create a firefly KLF7-WT luciferase vector. The putative miR-450b-3p binding sequence on KLF7 3′-UTR was then mutated to void miR-505 binding. The mutant (MUT) 3′-UTR was also inserted into p-miR-reporter to create a firefly KLF7-MUT luciferase vector. Human AGS and BGC-823 cells were transduced with NC mimic or miR-450b-3p mimic, then cross-transfected with KLF7-WT or KLF7-MUT for 48 h. After that, relative luciferase activities were measured using a Dual-Luciferase Reporter assay (Promega, USA) according to the manufacturer’s protocol.

### In vivo xenograft vectors

The Animal Ethics and Use Committee approved the tumor-forming experiment in nude mice. 8-week-old male nude mice were purchased from the animal center and randomly divided into two groups (5 in each group). The AGS cells with miR-450b-3p mimic were injected subcutaneously into the axilla of mice. Tumor size was monitored every 5 days; Then, after 6 weeks, the mice were sacrificed. The tumor volumes were calculated using the following formula: tumor volume = (width 2 × length)/2.

### Statistically analysis

Statistical analysis was performed using GraphPad Prism5 V5.01 software. Statistical differences between the two groups and multiple groups were analyzed using Student’s T test and one-way ANOVA, respectively. Independent experiments were repeated at least three times for each experiment, and the data were shown as averaged ± standard deviation. There were three levels of p < 0.05, p < 0.01 and p < 0.001 at the significance level, and p < 0.05 was considered statistically significant.

## Results

### miR-450b-3p was lowly expressed in GC tissues and cell lines

**Fig. 1:**
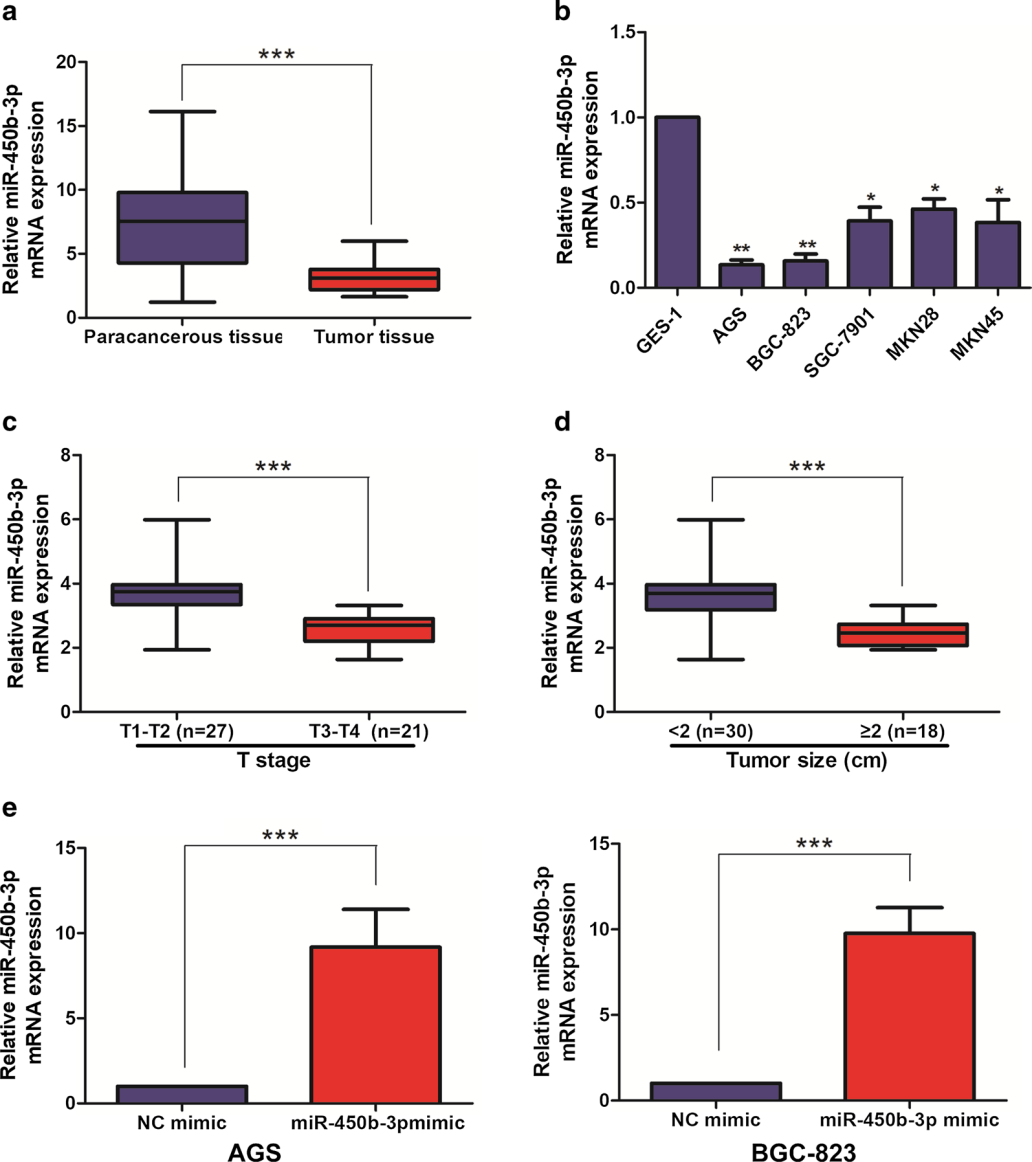
miR-450b-3p is lowly expressed in GC tissues and cell lines. **a** qRT-PCR was used to detect the expression level of miR-450b-3p in GC tissues and paracancerous tissues; **b** qRT-PCR was used to detect the expression level of miR-450b-3p in GC cell lines; **c** qRT-PCR was used to detect the difference expression of miR-450b-3p in tissue samples of GC patients with different pathological stage; **d** qRT-PCR was used to detect the difference expression of miR-450b-3p in tissue samples of GC patients with different tumor size; **e** qRT-PCR was used to verify the transfection efficiency of miR-450b-3p after transfection of NC mimic and miR-450b-3p mimic in AGS and BGC-823 cell lines. Data are mean ± SD, *p < 0.05, **p < 0.01, ***p < 0.001

The expression level of miR-450b-3p in 48 pairs of GC tumor tissue samples and corresponding paracancerous tissue ones was detected by qRT-PCR, and the results showed that miR-450b-3p expression was lower in GC tumor tissues than that in the corresponding paracancerous tissues (Fig. 1a). Meanwhile, miR-450b-3p was also found low-expression in GC cell lines, in particular AGS and BGC-823 cells, compared with GES-1, suggesting that miR-450b-3p might act as a cancer-suppressor gene in GC (Fig. 1b).

### miR-450b-3p was correlated with pathological stage and tumor size in GC patients

According to the expression level of miR-450b-3p, the above collected GC tumor tissue samples were divided into high-miR-450b-3p expression group and low-miR-450b-3p expression group. As shown in Table 1, the relationships between the expression of miR-450b-3p and Age, Gender, Pathological stage, Tumor size and Distant metastasis of GC patients were analyzed. The results showed that high expression of miR-450b-3p was positively correlated with Pathological stage (Fig. 1c) and Tumor size (Fig. 1d) in GC patients; But not with Age, Gender and Distant metastasis.

**Table 1:** Association of miR-450b-3p expression with clinicopathologic characteristics of gastric cancer

Parameters	Number of cases	miR-450b-3p expression		p-value
		High (%)	Low (%)	
Age (years)				0.971
< 60	21	11	10	
≥ 60	27	14	13	
Gender				0.386
Male	24	11	13	
Female	24	14	10	
T stage				*0.004*
T1-T2	27	19	8	
T3-T4	21	6	15	
Tumor size (cm)				*0.044*
< 2	30	19	11	
≥ 2	18	6	12	
Distance metastasis				0.157
No	28	17	11	
Yes	20	8	12	

Italic represents the significant difference

### Overexpression of miR-450b-3p inhibited cell proliferation in GC

**Fig. 2:**
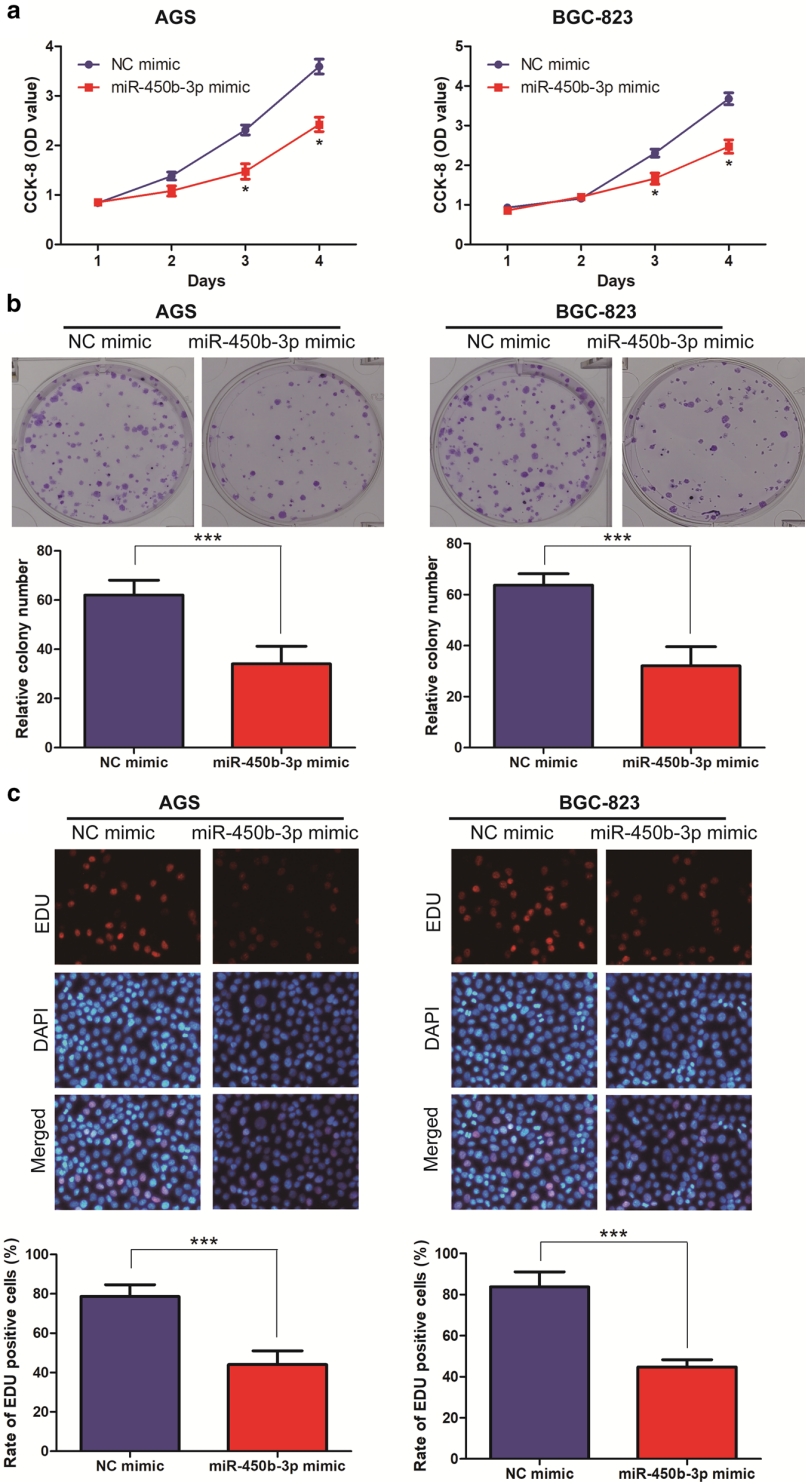
miR-450b-3p inhibited cell proliferation of GC. **a** CCK-8 assay detected cell proliferation after transfection of NC mimic and miR-450b-3p mimic in the GC cell lines; **b** Plate colony formation assay detected the clone forming ability after transfection of NC mimic and miR-450b-3p mimic in the GC cell lines (Magnification: ×10); **c**. EdU assay detected the proliferation ability after transfection of NC mimic and miR-450b-3p mimic in the GC cell lines (Magnification: ×40). Data are mean ± SD, *p < 0.05, ***p < 0.001

To investigate the biological functions of miR-450b-3p in GC cell lines, overexpression of miR-450b-3p vectors was constructed in AGS and BGC-823 cell lines. After transfecting NC mimic and miR-450b-3p mimic, qRT-PCR was performed to verify the transfection efficiency and the difference was shown statistically significant (Fig. 1e). After overexpression of miR-450b-3p, Cell proliferation, Plate colony formation and EdU assays were performed to assess the proliferation of miR-450b-3p in GC cells. As a result, the proliferation ability of GC cell lines with miR-450b-3p mimic was found significantly decreased when comparing with NC mimic (Fig. 2a–c).

### miR-450b-3p was bound to KLF7

**Fig. 3:**
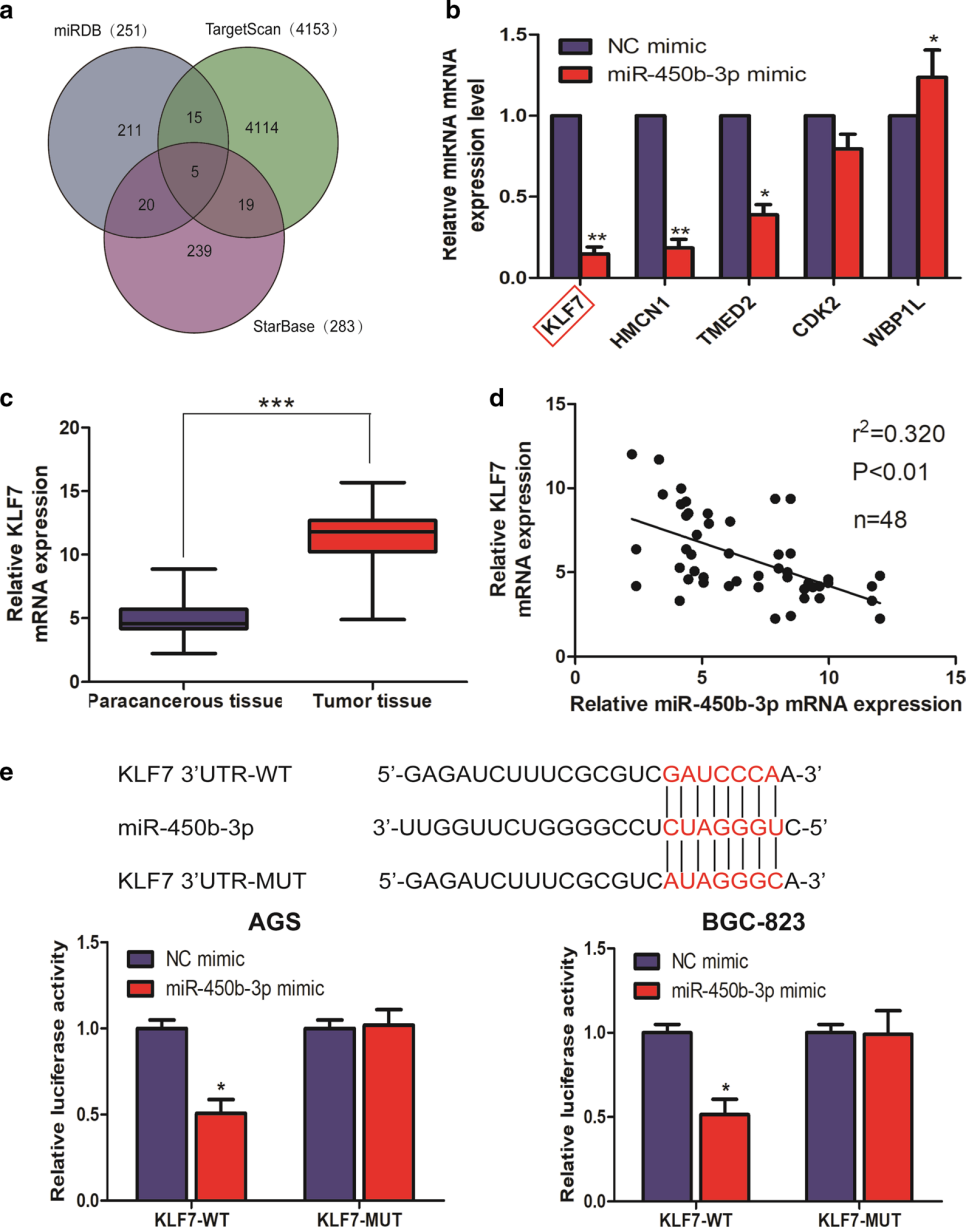
Interaction of miR-450b-3p and KLF7 in GC. **a** Bioinformatics analysis websites (miRDB, TargetScan and StarBase) suggested miR-450b-3p might be bound to KLF7; **b** qRT-PCR was used to detect the differential expression of the potential downstream target gene of miR-450b-3p in NC mimic and miR-450b-3p mimic, respectively; **c** qRT-PCR was used to detect the expression level of KLF7 in GC tissues and paracancerous tissues; **d** A significant negative correlation between miR-450b-3p and KLF7 expression in GC tissues; **e** Dual luciferase reporter assays demonstrated direct targeting of miR-450b-3p to KLF7. Data are mean ± SD, *p < 0.05, **p < 0.01, ***p < 0.001

Bioinformatics analysis websites (miRDB, TargetScan and StarBase) suggested miR-450b-3p might be bound to KLF7, HMCN1, TMED2 and so on (Fig. 3a). Subsequently, qRT-PCR revealed that compared with NC mimic, the expression level of KLF7 decreased most obviously in miR-450b-3p mimic (Fig. 3b). The results of qRT-PCR showed that KLF7 expression was higher in GC tumor tissues than that in the corresponding paracancerous tissues (Fig. 3c). Additionally, miR-450b-3p and KLF7 expression presented a significant negative correlation in GC tumor tissues (Fig. 3d). Luciferase reporter assay verified that miR-450b-3p could indeed combine with KLF7 through specific sequences (Fig. 3e).

### miR-450b-3p exactly regulated KLF7 to inhibit GC malignant progression

**Fig. 4:**
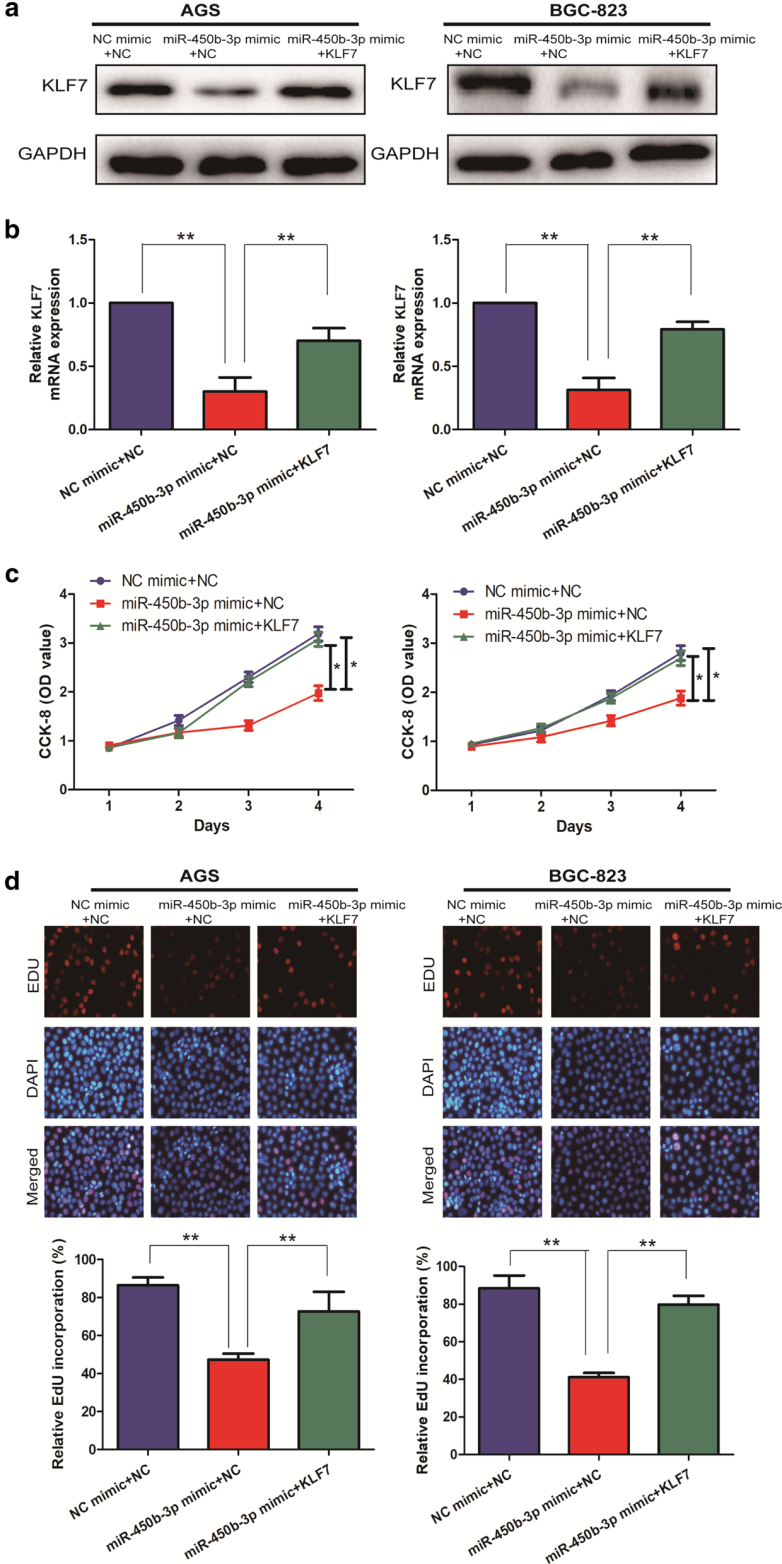
miR-450b-3p regulated the expression of KLF7 in GC cell lines. **a** The expression level of KLF7 in the co-transfected GC cell lines of miR-450b-3p and KLF7 was detected by Western Blotting; **b** The expression level of KLF7 in the co-transfected GC cell lines of miR-450b-3p and KLF7 was detected by qRT-PCR; **c** CCK-8 assay detected the cell proliferation in the co-transfected GC cell lines of miR-450b-3p and KLF7; **d** EdU assay was used to detect cell invasion and migration of GC cell lines after co-transfection of miR-450b-3p and KLF7 (Magnification: ×40). Data are mean ± SD, *p < 0.05, **p < 0.01

To further explore the specific regulatory mechanisms in which miR-450b-3p exactly regulated KLF7 to inhibit malignant progression of GC. Firstly, the overexpressed endogenous KLF7 was established in GC cell lines with miR-450b-3p mimic by transfecting Hep3B cells with a KLF7 overexpressing plasmid, or an empty overexpressing plasmid NC. Western Blotting and qRT-PCR demonstrated that GC cells transfected with miR-450b-3p mimic + KLF7 had significantly higher KLF7 expression levels than cells transfected with miR-450b-3p mimic + NC (Fig. 4a, b). Subsequently, overexpression of KLF7 was demonstrated to be able to counteract the effects of miR-450b-3p mimic on proliferation of GC cells by Cell proliferation and EdU assays (Fig. 4c, d). Therefore, these results revealed that miR-450b-3p inhibit the malignant progression of GC through modulating KLF7.

### miR-450b-3p upregulation suppressed GC in vivo tumorigenicity

**Fig. 5:**
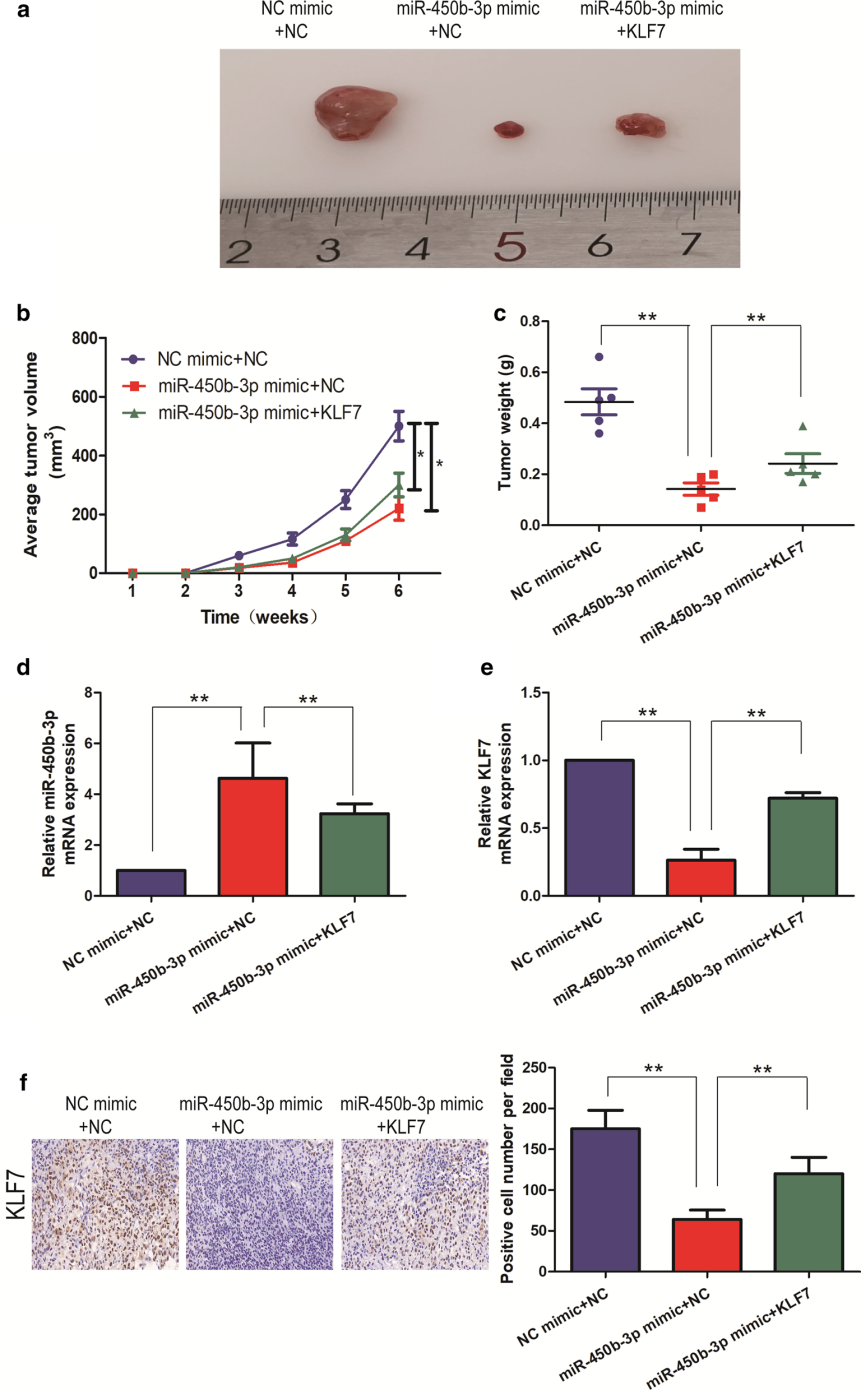
miR-450b-3p inhibited tumorigenic ability in nude mice. **a**, **b** Tumor volume growth curves were calculated for different nude mice after injection of NC mimic + NC, miR-450b-3p mimic + NC and miR-450b-3p mimic + KLF7, respectively; **c** Tumor weight growth curves were calculated after injection of NC mimic + NC, miR-450b-3p mimic + NC and miR-450b-3p mimic + KLF7, respectively; **d** qRT-PCR was used to detect the expression level of miR-450b-3p in the tumor-forming tissues of nude mice; **e** qRT-PCR was used to detect the expression level of KLF7 in the tumor-forming tissues of nude mice; **f** Immunohistochemistry was used to detect the expression level of KLF7 in the tumor-forming tissues of nude mice with AGS cell line (Magnification: ×40). Data are mean ± SD, *p < 0.05, **p < 0.01

In an in vivo tumorigenicity assay, NC mimic + NC, miR-450b-3p mimic + NC and miR-450b-3p mimic + KLF7 transduced AGS cells were subcutaneously inoculated into the abdominal compartments of athymic nu/nu mice for 6 weeks. The volumes of AGS xenografts were calculated weekly. It showed that, in vivo tumor growth was significantly suppressed by miR-450b-3p upregulation, and overexpression of KLF7 could reverse the inhibitory effect of tumor growth in nude mice with miR-450b-3p mimic (p < 0.05; Fig. 5a, b). Subsequently, we validated the reduction of weight in tumor-forming tissues of nude mice injected with miR-450b-3p mimic; as well as overexpression of KLF7 could reverse such effect (p < 0.05; Fig. 5c). In addition, compared with NC mimic + NC, miR-450b-3p expression dramatically increased in the tumor tissues of nude mice with miR-450b-3p mimic + NC by qRT-PCR; However, overexpression of KLF7 could decreased the level of miR-450b-3p (Fig. 5d). qRT-PCR found that the expression level of KLF7 was the opposite of the above (Fig. 5e). Immunohistochemistry showed that the level of miR-450b-3p mimic + KLF7-transduced AGS xenografts significantly increased than miR-450b-3p mimic + NC-transduced xenografts (Fig. 5F).

## Discussion

The etiology of GC is complex, and there is no clear conclusion on the pathogenesis except helicobacter pylori [10, 21]. Nowadays, the understanding of the pathogenesis and biological behavior of GC still has great limitations [5, 11]. The development of GC is a multi-factors process, influenced by a variety of biomolecules and regulated by signaling pathways [5]. Previous researches could only detect one or several genes at a time, and it is difficult to explain the whole process of tumor proliferation, invasion and metastasis [16]. With the progress of the Human Genome Project, researches on molecular level have been extensively conducted to detect the differential expression profile of tumor genes, which is of great significance to explore the molecular mechanism of the development of GC, and finding the molecular biomarkers in the early diagnosis and prognosis of GC [5, 7, 12].

Through epigenetic, transcriptional and post-transcriptional regulations, miRNAs have been recognized to widely participate in various biological processes [12, 16]. Furthermore, abnormally expressed miRNAs in tumors are well concerned [14, 16]. Functionally, miRNAs target on the corresponding mRNAs, and thus degrade them or inhibit their translation [14, 15]. It is estimated that over 30% of human genes and cellular processes are regulated or controlled by miRNAs [9, 16]. As a member of tumor-associated miRNAs family, miR-450b-3p is located on chromosome Xq23.11. Previous researches showed that miR-450b-3p could inhibit the malignant progress of breast cancer, hepatocellular carcinoma and so on by targeting the downstream related target genes [17, 18]. Therefore, we hypothesized that miR-450b-3p might regulate the malignant progress of GC. It was found that miR-450b-3p was expressed at low level in GC tumor tissues, compared with corresponding paracancerous ones. Besides, qRT-PCR showed that compared with GES-1, the expression level of miR-450b-3p was low among GC cell lines, in particular AGS and BGC-823 cells. Subsequently, it was also found that miR-450b-3p in tumor tissues of GC patients was positively correlated with pathological stage and tumor size in GC patients. Thus, the above results suggested that miR-450b-3p might act as a cancer-suppressor gene in GC. In order to further investigate the biological function of miR-450b-3p in GC cell lines, Cell proliferation, Plate colony formation and EdU assays were used to introduce that miR-450b-3p mimic could inhibit the proliferation ability of GC cell lines. This result also confirmed that miR-450b-3p expression might be related to cancer cell proliferation ability. The above results provided a theoretical basis for revealing the mechanism of GC development. Of course, the specific molecular mechanism of signal transduction in GC need to further study.

The cancer transcriptome is characterized by dysregulation of both protein-coding and noncoding transcripts [12, 15]. Competitive endogenous RNA (ceRNA) networks have been hypothesized, in which some functional RNAs can modulate each other’s transcription using miRNA response elements (MREs) [22]. Recently, several studies reported that miRNAs could antagonize the expression levels and biological function of mRNAs [12, 16]. Based on these findings, we hypothesized that miR-450b-3p might act as a ceRNA in GC and found that miR-450b-3p negatively regulated KLF7 expression by qRT-PCR and dual-luciferase reporter assays. In addition, qRT-PCR showed that compared with NC mimic, miR-450b-3p mimic could decreased the level of KLF7. In order to explore the associations between miR-450b-3p and KLF7 on the development of GC, the overexpression of KLF7 was found to reverse the proliferation ability of miR-450b-3p mimic on GC cells, thus promoting the malignant progression of GC.

## Conclusion

In summary, miR-450b-3p significantly down-regulated in GC tissues and cell lines, and was associated with the pathological stage and tumor size of GC patients. Meanwhile, miR-450b-3p inhibited GC cell proliferation via modulating KLF7.

## Data Availability

The datasets used in this study are available from the corresponding author upon reasonable request.
